# Alternative Models of Addiction

**DOI:** 10.3389/fpsyt.2015.00020

**Published:** 2015-02-13

**Authors:** Hanna Pickard, Serge H. Ahmed, Bennett Foddy

**Affiliations:** ^1^University of Birmingham, Birmingham, UK; ^2^University of Bordeaux: IMN CNRS UMR 5293, Bordeaux, France; ^3^New York University, New York, NY, USA

**Keywords:** drugs, addiction, disease, choice, compulsion, self-control, substance abuse, substance dependence

For much of the twentieth century, theories of addictive behavior and motivation were polarized between two models. The first model viewed addiction as a moral failure for which addicts are rightly held responsible and judged accordingly. The second model, in contrast, viewed addiction as a specific brain disease caused by neurobiological adaptations occurring in response to chronic drug or alcohol use, and over which addicts have no choice or control. As our capacity to observe neurobiological phenomena improved, the second model became scientific orthodoxy, increasingly dominating addiction research and informing public understandings of addiction. The articles in this research topic aim to move beyond the polarization between the competing moral and disease models of addiction.

In the opening article of this e-book “Addiction and Choice: Theory and New Data,” Heyman (1) examines new data on the ways that addicts recover, and argues that recovery from addiction is better predicted by a model in which addicts choose to use drugs, rather than one in which they are compelled to do so by a disease. This theme is echoed in other papers in this collection. Satel and Lilienfeld (2) in “Addiction and the Brain-Disease Fallacy” directly challenge the disease model, drawing on historical and clinical data to argue that addicts respond to incentives and use drugs for reasons, and so addictive behavior must be understood as a choice. In “Intertemporal Bargaining in Addiction,” Ainslie (3) reprises his large body of work on the inherent weaknesses of the human capacity for choice, exploring its relevance to questions of the nature of responsibility and our justification in holding addicts accountable for addictive behavior and its consequences.

Other authors in this volume seek to understand the ways in which these choices can be pathologically impaired by addiction. For example, Dill and Holton’s (4) article “The Addict In Us All” contrasts ordinary choices with what they call the “incentive salience” choices, which are typical in addictive consumption, and involve extreme cravings for drugs and strong motivation to consume, even when consumption is neither experienced nor judged as desirable. Henden et al. (5) in “Addiction: Choice or Compulsion?” chart a course between the moral and disease model by arguing that addictive behavior can be labeled both voluntary and compulsive, if this is understood as involving repeated decisions, which can lead to maladaptive and self-destructive behavioral outcomes.

The importance of negative outcomes to understanding addiction is emphasized by both Levy (6) and Wakefield and Schmitz (7). In “Addiction is Not a Brain Disease (And it Matters),” Levy argues that while addiction does produce neurological dysfunction, this is not enough to make it a disease. In Levy’s eyes, disease necessarily involves impairment, and impairment must be understood relative to the social and practical context in which addicts live. In contrast, Wakefield and Shmitz’s article “How Many People Have Alcohol Use Disorders?” points out that by focusing too heavily on the negative health correlates of chronic alcohol use, DSM-IV and DSM-V diagnostic criteria risk diagnosing non-addicts suffering the ill-effects of long-term use with an addictive “disease.”

The social and practical context of addiction is also emphasized by other authors. It has long been known that cocaine-addicted rats will forego cocaine if offered alternative goods, such as sugar or saccharin. Zernig et al.’s (8) article “Dyadic Social Interaction as an Alternative Reward to Cocaine” presents data demonstrating that, in certain experimental conditions, rats will also forego cocaine for the opportunity of same-sex snuggling. Overall, these findings further show that drug choices are not determined by the ability of a drug to directly activate and/or sensitize the reinforcing and incentive salience neuronal pathways in the brain. This conclusion is consistent with the general evolutionary view of drug use advanced by Hagen et al. (9). Their article notes that the most currently widely used drugs, like cannabis, cocaine, and nicotine, are originally plant chemical defenses that evolved to deter consumption. To adapt, animals are likely to have evolved internal protective mechanisms allowing them to use drugs in a controlled manner.

In “The Shame of Addiction,” Flanagan (10) argues that the first-personal experience of shame – typically a social and moral emotion – is central to understanding human addiction and the motivation addicts have to heal, but that shame can be distinguished from blame, allowing addiction to be conceived as an aspect of personal agency without returning to the moral model. In the final article of this e-book, Heyman et al. (11) present data suggesting that years spent in school is a key predictor of illicit drug use, after controlling for IQ and impulsivity, suggesting not only a potential social cause of addiction but also, equally, a social solution.

The e-book also contains articles exploring the classification of addiction and its potential status as a natural kind, the role and extent of pleasure in explanations of addiction, and various more unusual forms of addiction, such as workaholism, which we naturally characterize as involving a need for control, as opposed to involving a loss of control.

Considered as a whole, the articles in this volume demonstrate that we can conceptualize addiction as choice, while avoiding both the scylla of moralization and the charybda of brain disease. By doing so, they emphasize the need to give equal attention and weight to the historical, contextual, and biological factors that are significant in addiction, to move forward in understanding and responding to the problem. Addiction as choice may thus offer a unifying and integrative framework for future research in the field.

## Conflict of Interest Statement

The authors declare that the research was conducted in the absence of any commercial or financial relationships that could be construed as a potential conflict of interest.
